# Nitric Oxide Modulation by Folic Acid Fortification

**DOI:** 10.3390/antiox9050393

**Published:** 2020-05-07

**Authors:** Junsei Taira, Takayuki Ogi

**Affiliations:** 1 Department Bioresources Engendering, Okinawa College, National Institute of Technology, 905 Henoko, Nago, Okinawa 905-2192, Japan; 2 Okinawa Industrial Technology Center, 12-2 Suzaki, Uruma, Okinawa 904-2234, Japan; ogitkyuk@pref.okinawa.lg.jp

**Keywords:** folic acid, nitric oxide, neural tube defects, RAW264.7 cells, NOR3, ESR, LC/MS

## Abstract

Folic acid (FA) can be protected the neural tube defects (NTDs) causing nitric oxide (NO) induction, but the alleviation mechanism of the detailed FA function against NO has not yet been clarified. This study focused on elucidation of the interaction of FA and NO. FA suppressed nitrite accumulation as the NO indicator in lipopolysaccharide (LPS)-stimulated RAW264.7 cells, then the expression of the *i*NOS gene due to the LPS treatment was not inhibited by FA, suggesting that FA can modulate against NO or nitrogen radicals. NOR3 (4-ethyl-2-hydroxyamino-5-nitro-3-hexenamide) as the NO donor was used for evaluation of the NO scavenging activity of FA. FA suppressed the nitrite accumulation in a dose-dependent manner. To confirm the reaction product of FA and NO (FA-NO), liquid chromatography–mass spectrometry (LC/MS) was used to measure a similar system containing NOR3 and FA, and then detected the mass numbers of the FA-NO as *m/z* 470.9 (M + H)^+^ and *m/z* 469.1 (M − H)^−^. In addition, the adducts of the FA-NO derived from ^14^NO and ^15^NO gave individual mass numbers of the isotopic ratio of nitrogen for the following products: FA-^14^NO, *m/z* 471.14 (M + H)^+^; *m/z* 469.17 (M − H)^−^ and FA-^15^NO, *m/z* 472.16 (M + H)^+^; *m/z* 470.12 (M − H)^–^. To clarify the detailed NO scavenging action of FA, an electron spin resonance (ESR) study for radical detecting of the system containing carboxy-PTIO (2-(4-carboxyphenyl)-4,4,5,5-tetramethylimidazoline-1-oxyl-3-oxide) as an NO detection reagent in the presence of NOR3 and FA was performed. The carboxy-PTI (2-carboxyphenyl-4,4,5,5-tetramethylimidazoline-1-oxyl) radical produced from the reaction with NO reduced in the presence of FA showing that FA can directly scavenge NO. These results indicated that NO scavenging activity of FA reduced the accumulation of nitrite in the LPS-stimulated RAW264.7 cells. The NO modulation due to FA would be responsible for the alleviation from the failure in neural tube formation causing a high level of NO production.

## 1. Introduction

The falling of neural tube defects (NTDs) in neural tube formation during early embryogenesis includes anencephaly, exencephaly, and spina bifida. Folic acid (FA) is known as a dietary supplement that can be prevent NTDs involving failure of the neural tube (NT) closure in the developing embryo, especially spina bifida and anencephaly in the periconceptional period [1].

Based on this information, the mandatory FA fortification has been associated with a decline in NTD prevalence in many countries [2,3,4]. A more recent study demonstrated that microglia activation, including the disruption of the endogenous inhibitory system (CD200-CD200R), contributes to injury in spina bifida aperta after birth [5]. Previous study indicated that a moderate level of nitric oxide (NO) and nitric oxide synthase (NOS) play a critical role in normal embryonic development [6]. Physiological concentrations of NO modulate carbon flow through the folate pathway, that is, NO inhibits methionine synthase (MS) involving the interference transfer of the methyl group from the methyl donor, 5-methyl-tetrahydrofolate (5mTHF), to homocysteine during methionine production [7].

In previous studies, the direct effect of FA against NO produced by the NO donor, *S*-nitroso-*N*-acetyl-penicillamine (SNAP), on the process of NT closure in the chick embryo ex ovo was examined. NOS involves high NO levels due to the SNAP treatment of the inactivated MS and its activity can only be rescued by FA or vitamin B12, but not by the NOS inhibitor [8,9]. Although FA or vitamin B12 can prevent the NTDs causing the NO induction, the detailed prevention mechanism due to the FA relation to NO has not yet been clarified. Therefore, this study focused on the interaction of FA and NO, and this article describes how FA can directly scavenge NO involved in NTDs.

## 2. Materials and Methods

### 2.1. Reagents

4-Ethyl-2-hydroxyamino-5-nitro-3-hexenamide (alternate name: NOR3) and 2-(4-carboxyphenyl)-4,4,5,5-tetramethylimidazoline-1-oxyl-3-oxide (alternate name: carboxy-PTIO) were purchased from Dojindo Molecular Technique Inc. (Kumamoto, Japan). Folic acid (FA), l-arginine, interferon-γ (IFN-γ) and lipopolysaccharide (LPS) were obtained from the FUJI Firm Wako Pure Chemical Corporation (Osaka, Japan). Dulbecco’s modified Eagle’s medium (DMEM) and fetal bovine serum (FBS) were obtained from Gibco BRL (Grand Island, NY, USA).

### 2.2. Cell Culture

Raw264.7 cells (mouse macrophages, American type culture collection) were cultured in DMEM in 10% FBS, 100 U/mL penicillin and 100 µg/mL streptomycin at 37 °C in a 5% CO_2_ atmosphere.

### 2.3. Nitrite Assay on RAW264.7 Macrophages

RAW264.7 macrophages in a 96-well microplate were treated with LPS (100 ng/mL), l-arginine (2 mM), and IFN-γ (100 U/mL) with or without the various concentrations of FA (25–200 µM). After culturing for 16 h, the nitrite production as an NO indicator in the medium, was determined by the Griess method as previously reported [10,11].

### 2.4. iNOS Gene Expression

Reverse transcriptase-polymerase chain reaction (RT-PCR) was carried out according to previously described procedures [12]. Briefly, the LPS-stimulated RAW264.7 macrophages on a 12-well microplate (2.5 × 10^6^ cells/mL) were treated with the FA (200 µM). The total RNA from cells was isolated from the cell lysate and the amplification of the cDNA was performed by the *i*NOS primers: 5`-CCT TGT TCA GCT ACG CCT TC-3`and 5`-CTG AGG GCT CTG TTG AGG TC-3` using PCR (GeneAmp^®^ PCR System 9700, Applied Biosystems, Waltham, MA. USA). The PCR product of cDNA (100 ng/µL) was loaded on a DNA chip (Agilent DNA 1000 kit, Agilent Technologies, Santa Clara, CA, USA) and the electrophoresis was performed by a micro DNA analyzer (Agilent 2100 Bioanalyzer, Agilent Technologies, Santa Clara, CA, USA).

### 2.5. Nitric Oxide (NO) Inhibitory Action

NOR3 as the NO donor was used in the presence of various concentrations of FA (10–200 µM) as previously reported [13]. The reaction mixture containing NOR3 (200 µM), with or without FA in phosphate-buffered saline (PBS) solution, was incubated at room temperature for 60 min. The nitrite accumulation in the reaction mixture was determined using previously reported procedures [10,11]. In addition, the reaction mixture was analyzed by liquid chromatography–mass spectrometry (LC/MS) equipment. The reaction product of FA and NO was measured by LC/MS using a photodiode array detector (Quattromicro API triple-quadruple mass analyzer, Waters Corp., Milford, MA, USA) and monitored at 275 nm on a reversed-phase chromatographic column, YMC Pro C18 (i.d. 3 mm × 10 cm, YMC Co., Ltd., Kyoto, Japan) at 40 °C. The mobile phase consisting of 1% formic acid and acetonitrile solvent (10%) was carried out at the flow rate of 0.34 mL/min by a linear gradient to 10%, 30% and 100% for 2 min, 3 min and 7 min. The mass spectra were measured under the following conditions: electrospray ionization (ESI) at the cone voltages of 17 volts for the positive ion mode and at 21 volts for the negative ion mode.

### 2.6. Electron Spin Resonance (ESR) Measurement

The NO scavenging ability of FA was examined in the presence of the carboxy PTIO as the NO detection reagent [14]. The reaction mixture of FA (200 µM), NOR3 (100 µM) and carboxy-PTIO (50 µM) was prepared in PBS and incubated at room temperature for 15 min. An electron spin resonance (ESR) measurement was performed by an ESR spectrometer (JES-FR30, JEOL, Ltd., Tokyo, Japan) operating at the X-band with the modulation frequency of 100 kHz. The reaction mixture was transferred to the capillary (100 × 1.1 mm i.d. (inner diameter)., Drummond Scientific Co., Broomall, PA, USA) and placed in a quartz cell (270 mm long, 5 mm i.d., JEOL DATUM, Ltd., Tokyo, Japan). The ESR signal was measured at 9.4 GHz resonant frequency the following conditions: microwave power; 4 mW; modulation width, 0.1 mT; gain, 500; scan time, 1 min; time constant, 0.3 s.

### 2.7. Reaction Product of Folic Acid (FA) and NO

The reaction products of FA and NO were prepared as previously reported [11,15]. Milli-Q water (200 mL) was degassed using an ultrasonic device under reduced pressure for 30 min, subsequently a dry nitrogen gas was bubbled for 30 min in a nitrogen gas-filled glove box (762 × 450 × 478 mm, AS-600PC, AS ONE corp., Osaka, Japan). The status of deoxygenation in the aqueous solution was confirmed using an oximeter (MDS-2C, Marubishi Bioengineering Co., Ltd., Tokyo, Japan). This deoxygenated aqueous solution (1 mL) containing FA (4.4 mg), Na_2_S_2_O_4_ (20 mg), Na^14^NO_2_ or Na^15^NO_2_ (40 mg) was incubated in a capped vial at room temperature for 1 h under the anoxic conditions in the glove box. While the similar reaction mixture as control was prepared under the aerobic conditions. Then, the product of NO (FA-^14^NO and FA-^15^NO) was immediately analyzed by LC/MS equipment. The reaction product of FA and NO was measured by LC/MS using a photodiode array detector (Xevo-TQD triple-quadrupole mass analyzer, Waters, MA, USA) and monitored at 275 nm on a reversed-phase chromatographic column, Acquity UPLC BEH C18 (i.d. 2.1 mm × 3 cm, 1.7 μm, Waters) at 40 °C. The mobile phase consisting of 0.1% formic acid aqueous solution (100%) and acetonitrile solvent containing 0.1% formic acid was carried out at the flow rate of 0.40 mL/min by a linear gradient to 100%, 5%, 5% for 0.5 min, 3.5 min, 5min. These mass spectra were measured under the following conditions: ESI at the cone voltages of 30 volt in both the positive and negative ion mode.

## 3. Results

### 3.1. NO Inhibitory Action

**Figure 1:**
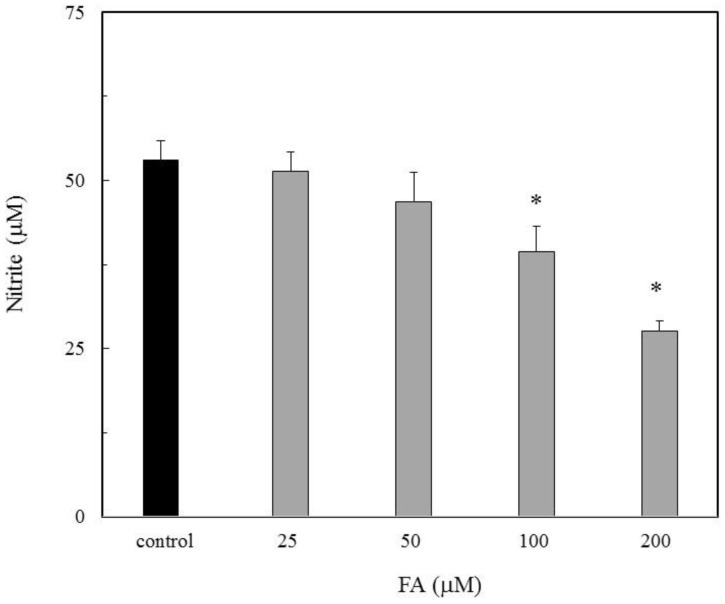
Inhibition of folic acid (FA) for nitric oxide (NO) production in lipopolysaccharide (LPS)-stimulated RAW264.7 macrophages. The various concentrations of FA (25, 50, 100 and 200 µM) were evaluated for the NO production in the LPS-stimulated RAW264.7 macrophages. Data are expressed as mean ± standard deviation (SD) and the significant difference was analyzed by the Student’s *t*-test. *p < 0.01 indicates significant difference from the control.

The NO inhibitory action due to FA was evaluated for the NO production in the LPS-stimulated RAW264.7 cells. The nitrite accumulation as the NO indicator increased in the LPS treated cells. When FA was placed in the cells, the nitrite accumulation was inhibited in a dose dependent manner (Figure 1). A previous study showed a similar result that FA inhibited cytotoxicity with apoptosis due to NO [16]. This result indicated that FA has an inhibitory action on the NO production.

### 3.2. NO Scavenging Activity

**Figure 2:**
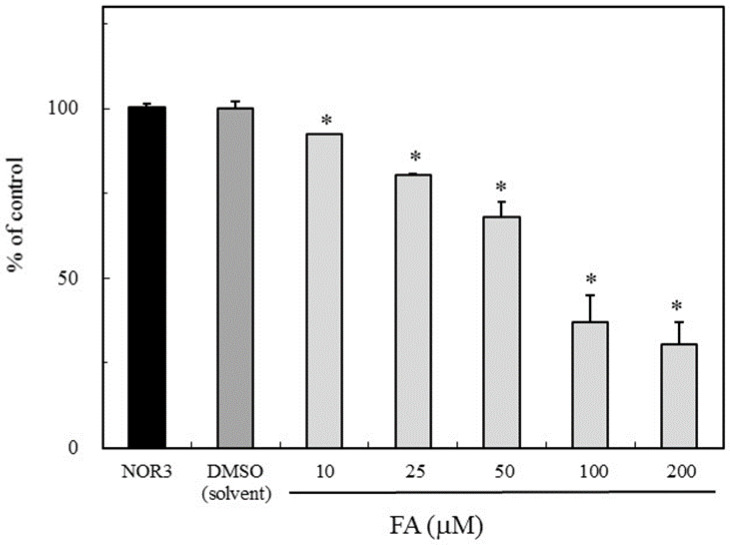
Inhibition of nitrite accumulation due to folic acid (FA). The reaction mixture containing NOR3 (4-ethyl-2-hydroxyamino-5-nitro-3-hexenamide) (200 µM) as the NO donor with or without FA (10, 25, 50, 100 and 200 µM) in phosphate-buffered saline (PBS) solution was incubated at room temperature for 60 min. The nitrite level was used as the NO indicator. Data are expressed as mean ± SD, and the significant difference was analyzed by the Student’s *t*-test. *p < 0.01 indicates significant difference from NOR3 without the test compounds.

The NO scavenging activity due to FA was evaluated using NOR3 as the NO donor. The nitrite accumulation as the NO indicator was examined with or without FA. As shown in Figure 2, FA suppressed the nitrite accumulation in a dose-dependent manner. This result suggested that FA has a potential NO or nitrogen radical scavenging activity. Therefore, the NO scavenging ability due to FA would be responsible for the suppression of the NO production in the LPS-stimulated RAW264.7 cells (Figure 1).

### 3.3. Suppression of iNOS Gene Expression

**Figure 3:**
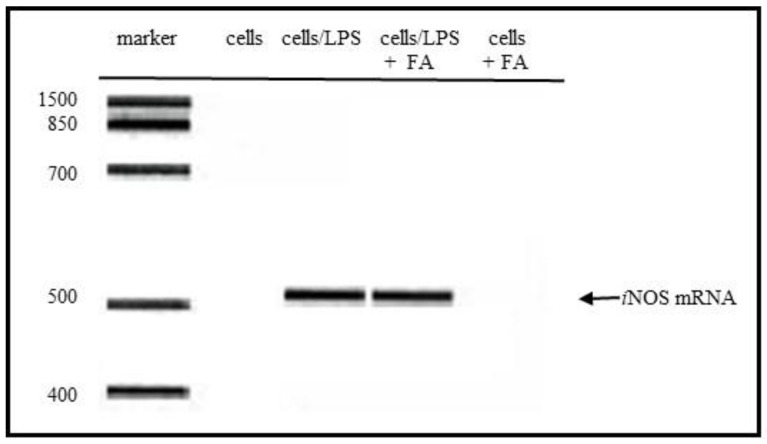
Inhibitory effect of folic acid (FA) for LPS stimulated *i*NOS mRNA expression in LPS-stimulated RAW264.7 macrophages. An *i*NOS mRNA gene expression was induced in the LPS-stimulated RAW264.7 cells. The *i*NOS gene expression in the cells was examined with or without FA (200 µM). Cells, cells without treatment; cells/LPS, cells treated with LPS; cells/LPS + FA, cells treated with LPS and cells + FA, cells treated with FA.

The *i*NOS mRNA gene expression was induced in the LPS-stimulated RAW264.7 cells. The *i*NOS gene expression in the cells was examined with or without FA. As shown in Figure 3, the *i*NOS gene expression by the LPS treatment was not suppressed by the FA treatment. This result suggested that FA can directly scavenge NO or nitrogen radicals which would be responsible for the suppression of the NO production in the LPS-stimulated cells (Figure 1).

### 3.4. Determination of FA-NO by Liquid Chromatography–Mass Spectrometry (LC/MS)

**Figure 4:**
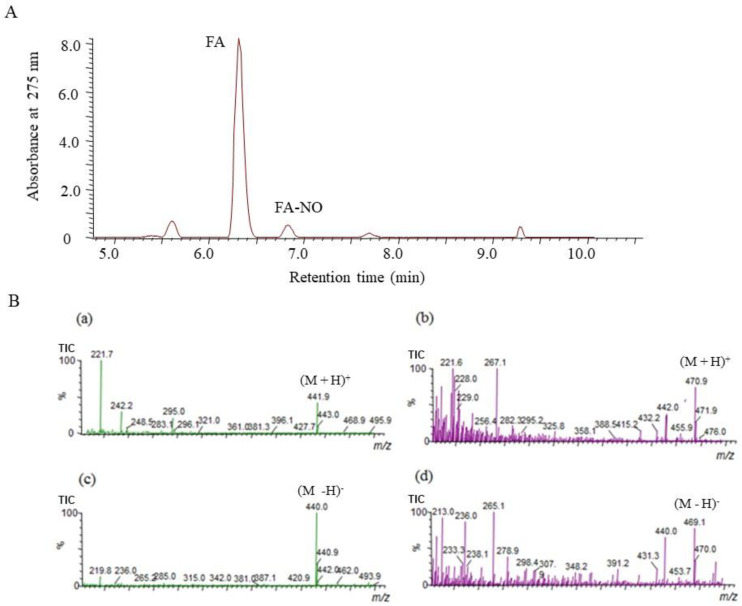
Liquid chromatography–mass spectrometry (LC/MS) chromatogram obtained from reaction mixture containing NOR3 and folic acid (FA). The LC/MS was carried out under the analytical conditions as described in the text. (**A**) Each peak of the high-performance liquid chromatography (HPLC) chromatogram indicated FA and its reaction product with NO, FA-NO. (**B**) These mass spectra indicated (**a**) FA, *m/z* 441.9 (M + H)^+^ and (**b**) FA-NO, *m/z* 470.9 (M + H)^+^ in the positive ion mode and (**c**) FA, *m/z* 440.0 (M − H)^−^ and (**d**) FA-NO, *m/z* 469.1 (M − H)^−^ in the negative ion mode of mass spectrometry.

To confirm the reaction product of FA and NO (FA-NO), LC/MS was used to measure in the reaction mixture containing NOR3 and FA. The high-performance liquid chromatography (HPLC) chromatogram of the FA-NO produced in the presence of FA and NOR3 indicated in Figure 4. The mass spectrum of the reaction products was detected in both the positive and negative ion mode. The mass numbers of *m/z* 470.9 (M + H)^+^ and *m/z* 469.1 (M − H)^−^ indicated that the FA-NO was produced by the interaction of FA and NO.

### 3.5. Products of FA-^14^NO and FA-^15^NO

**Figure 5:**
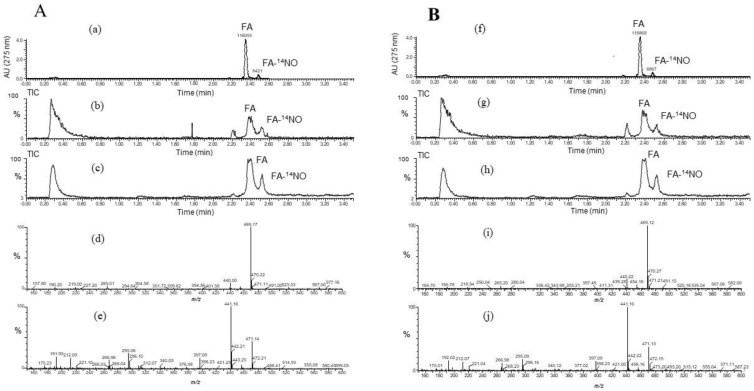
LC/MS chromatogram obtained from the reaction mixture containing folic acid (FA), Na_2_S_2_O_4_ and Na^14^NO_2_ under the conditions of (**A**) aerobic and (**B**) anoxia. The LC/MS was carried out under the analytical conditions as described in the text. Each peak of LC/MS indicated FA and its reaction product with NO, FA-NO. The HPLC chromatogram indicated FA and its reaction product with NO under the conditions of **A** (**a**) aerobic and **B** (**f**) anoxia. The TIC (total ion chromatogram) and its mass spectra of FA–^14^NO indicated in the negative and positive ion modes as follows. The aerobic conditions; the TIC of (**b**) and (**c**) for (**d**) *m/z* 469.17 (M − H)^−^ and (**e**) *m/z* 471.14 (M + H)^+^, and the anoxic conditions; the TIC of (**g**) and (**h**) for (**i**) *m/z* 469.12 (M − H)^−^ and (**j**) *m/z* 471.13 (M + H)^+^.

**Figure 6:**
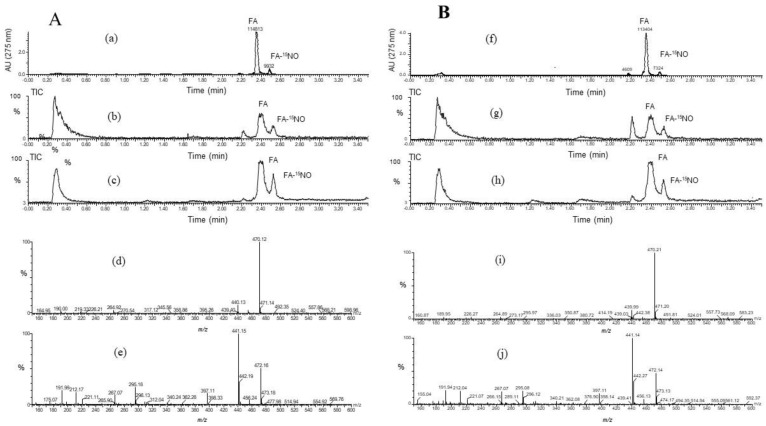
LC/MS chromatogram obtained from the reaction mixture containing folic acid (FA), Na_2_S_2_O_4_ and Na^15^NO_2_ under the conditions of (**A**) aerobic and (**B**) anoxia_._ The LC/MS was carried out under the analytical conditions as described in the text. Each peak of LC/MS indicated FA and its reaction product with NO, FA-NO. The HPLC chromatogram indicated FA and its reaction product with NO under conditions that were **A** (**a**) aerobic and **B** (**f**) anoxia. The TIC (total ion chromatogram) and its mass spectra of FA-^14^NO indicated in the negative and positive ion modes as follows. the aerobic conditions; the TIC of (**b**) and (**c**) for (**d**) *m/z* 470.12 (M − H)^−^ and (**e**) *m/z* 472.16 (M + H)^+^, and the anoxic conditions; the TIC of (**g**) and (**h**) for (**i**) *m/z* 470.21 (M − H)^−^ and (j) *m/z* 472.14 (M + H)^+^.

To confirm the ability of the FA scavenging of NO, the product of FA-NO derived from ^14^NO and ^15^NO was prepared, and then each mass spectrum was measured by LC/MS. As shown in Figure 5 and Figure 6, each reaction product of ^14^NO and ^15^NO with FA under the conditions of aerobic and anoxia was as follows; FA-^14^NO, *m/z* 469.17 (M − H)^−^; *m/z* 471.1 4 (M + H)^+^ for the aerobic conditions and *m/z* 469.12 (M − H)^−^; *m/z* 471.13 (M + H)^+^ for the anoxic conditions (Figure 5) and also FA-^15^NO, *m/z* 470.12 (M − H)^−^; *m/z* 472.16 (M + H)^+^ for the aerobic conditions and *m/z* 470.21 (M − H)^−^; *m/z* 472.14 (M + H)^+^ for the anoxic conditions (Figure 6). The individual mass number of the FA-NO was clearly distinguished by the difference in the isotopic ratio of nitrogen. In addition, the product of FA-NO in the anoxic conditions was similar yield to that of the aerobic conditions. These results supported the assertion that FA has NO scavenging ability, resulting in the suppression of NO production in the LPS-stimulated RAW264.7 cells (Figure 1).

### 3.6. FA Scavenging NO by ESR Study

**Figure 7:**
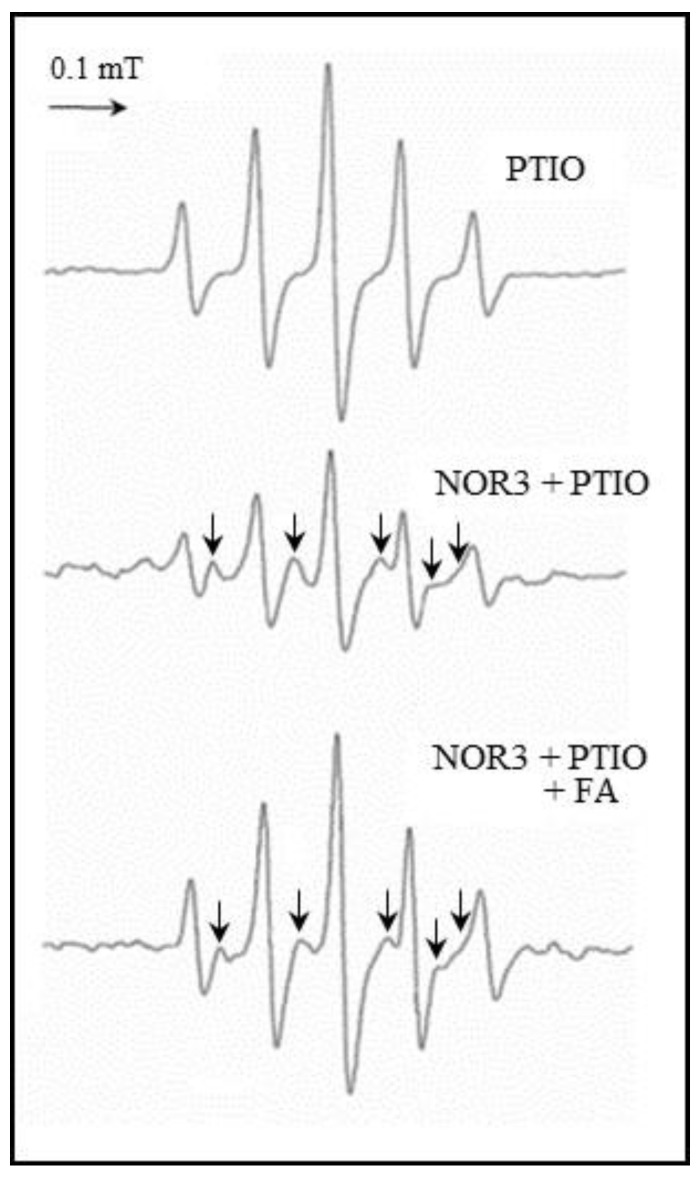
NO savenging activity of folic acid (FA). Electron spin resonance (ESR) spectrum obtained from the reaction mixture containing the carboxy-PTIO (2-phenyl-4,4,5,5-tetramethylimidazoline-1-oxyl-3-oxide, 200 µM) as the NO detection reagent in the presence of NOR3 (200 µM) with or without FA (200 µM). The NO released from NOR3 was detected by carboxy-PTIO, then produced a carboxy-PTI (2-phenyl-4,4,5,5-tetramethylimidazoline-1-oxyl) radical as indicated by the arrows.

It is known that nitronyl nitroxide carboxy-PTIO as an NO detection reagent reacts with NO, generate an imino nitroxide, carboxy-PTI radical and NO_2_ [17,18]. To clarify the NO scavenging action of FA, an ESR study was performed on the system containing the carboxy-PTIO in the presence of NOR3 and FA. The NO released from NOR3 was detected by carboxy-PTIO, then produced a carboxy-PTI radical as indicated by the arrows in Figure 7. The carboxy-PTI radical was reduced when FA was present in the system, indicating that FA can directly scavenge NO.

## 4. Discussion

The significance of FA in the prevention of neural tube defects (NTDs) involving the failure of NT closure in the developing embryo is well recognized. NO had been shown to be able to induce NTDs in rat embryos, and biochemical studies showed that NO inhibits methionine synthase (MS) [6,7]. In a previous study, the direct effect of NO produced by the NO donor SNAP on the process of NT closure in the chick embryo ex ovo. was evaluated and the high NO levels by the SNAP treatment inhibited the MS in the methyl transfer reaction of cofactor B12 [8,9]. The alleviation mechanism due to the FA treatment was speculated to be the interference with one-carbon flow through the folate pathway [8]. Previous studies showed that 5-methyl-tetrahydrofolate (5-mTHF), the primary circulating metabolite of FA, improves the endothelial NO synthase (*e*NOS) coupling cofactor tetrahydrobiopterin (BH_4_), indicating that the NOS activity regulates the MS activity in the process of NT closure [8]. On the other hand, when the BH_4_ is limited, the uncoupled NOS produces the superoxide radical (O_2_^−^) rather than NO, then produces peroxynitrite by the reaction between O_2_^-^ and NO which causes the endothelial dysfunction [19]. FA has the ability of O_2_^−^ scavenging, which may protect or improve the endothelial function [20,21,22,23]. However, the detailed relationship between FA and NO has not yet been clarified. In this study, the effect of FA on the NO production in the LPS-stimulated RAW264.7 cells was examined. The nitrite accumulation in the LPS treatment cells decreased in the presence of FA (Figure 1). An inflammatory cytokine or proinflammatory cytokine including interleukin-1 (IL-1), interleukin-12 (IL-12), and interleukin-18 (IL-18), TNF-α, interferon γ (IFNγ), and granulocyte-macrophage colony stimulating factor (GM-CSF) stimulate the Janus kinase (Jak) and signal transducer and activator of transcription (STAT) pathway (JAK-STAT pathway) as a key role in signal pathways activated by growth factors and cytokines. Expression of *i*NOS and the production of NO in response to LPS/IFN γ are increased through JAK/STAT signaling [24,25]. While the expression of the *i*NOS gene due to the LPS/ IFNγ treatment was not inhibited by the FA treatment, thus the reduction NO due to FA will not be through the JAK-STAT pathway. It is suggested that FA may directly regulate against NO or producing nitrogen radicals during the NO oxidation (Figure 2). The NO-scavenging ability of FA was examined in the reaction with NOR3 as the NO donor. FA suppressed the nitrite accumulation in a dose-dependent manner suggesting that FA provides a direct scavenging ability against NO or nitrogen radicals. The result first showed another possible function of FA which can modulate the nitrogen radicals including NO. Although FA could not suppress the *i*NOS gene expression, the nitrogen radical scavenging function due to FA could suppress for the NO production in the LPS-stimulated cells (Figure 3). To better clarify the function of FA, the FA scavenging NO adduct in the reaction mixture was detected by LC/MS. The mass numbers of *m/z* 470.9 (M + H)^+^ and *m/z* 469.1 (M − H)^−^ were determined as the adduct of the FA scavenging NO (FA-NO). To obtain more detailed evidence, the FA-NO derived from ^14^NO and ^15^NO was measured in both the positive and negative ion mode, and each product of the ^14^NO or ^15^NO reaction with FA gave the following mass numbers: FA-^14^NO, *m/z* 471.14 (M + H)^+^; *m/z* 469.17 (M − H)^−^ and FA-^15^NO, *m/z* 472.16 (M + H)^+^; *m/z* 470.12 (M − H)^−^ (Figure 5A and Figure 6A). The individual mass number of the FA-NO was clearly distinguished by the difference in the isotopic ratio of nitrogen, resulting in the product by the FA reaction with NO. NO reacts with oxygen giving rise to the formation of NO_2_ and potentially N_2_O_3_. To avoid the reactions, the FA reaction with NO in the anoxic conditions was examined, resulting in the product of FA-NO being of a similar yield to that of the aerobic conditions (Figure 5B and Figure 6B). This result suggested that the reactions of FA with NO give rise to folic acid nitrosation.

To clarify the direct NO scavenging action due to FA, an ESR study was performed on the system containing carboxy-PTIO as the NO detection reagent in the presence of NOR3 and FA. The carboxy-PTI radical was then produced from the reaction with NO reduced in the presence of FA (Figure 7). This result strongly supported the assertion that FA can directly scavenge NO and the function of FA suppressed the NO production in the LPS-stimulated cells.

FA can prevent the NTDs causing the NO induction which inhibits MS involving the interference transfer of the methyl group from the 5mTHF to homocysteine in methionine production. This study proposes another alleviation mechanism whereby the NO modulation due to the FA direct scavenging of NO contributes to alleviation from the failure in the NT formation causing the high level of NO production.

## 5. Conclusions

This study first demonstrated that FA can directly scavenge NO, resulting in the reduction of the accumulation of nitrite in the LPS-stimulated RAW264.7 cells. In addition, the NO modulation due to FA may contribute to alleviation from the failure in neural tube formation causing the high level of NO production.
